# Structural insights into a regulatory mechanism of FIR RRM1–FUSE interaction

**DOI:** 10.1098/rsob.230031

**Published:** 2023-05-31

**Authors:** Xiaomin Ni, Andreas C. Joerger, Apirat Chaikuad, Stefan Knapp

**Affiliations:** ^1^ Institute of Pharmaceutical Chemistry, Goethe-University Frankfurt, Max-von-Laue-Str. 9, 60438 Frankfurt, Germany; ^2^ Structural Genomics Consortium (SGC) and Buchmann Institute for Molecular Life Sciences, Max-von-Laue-Str.15, 60438 Frankfurt, Germany

**Keywords:** RNA recognition motif (RRM), RNP2 motif, nucleotide configuration

## Abstract

FUBP-interacting repressor (FIR) is a suppressor of transcription of the proto-oncogene MYC. FIR binds to the far upstream element (FUSE) of the MYC promoter. Competition of FIR with FUSE-binding protein 1 (FUBP1) is a key mechanism of MYC transcriptional regulation. To gain insights into the structural mechanisms regulating FIR DNA interaction, we determined the crystal structure of two FIR RRM domains (RRM1-2) with single-stranded FUSE DNA sequences. These structures revealed an ability of the RRM domain to recognize diverse FUSE regions through distinct intermolecular interactions and binding modes. Comparative structural analyses against available RRM-ssDNA/RNA complexes showed that the nucleotide configurations in FIR were similar to those in other RRMs that harbour a tyrosine at the conserved aromatic position in the RNP2 motif (Y-type RRM), but not those with a phenylalanine (F-type RRM). Site-directed mutagenesis experiments demonstrated that a single substitution, Y115F, altered the binding affinities of oligonucleotides to FIR RRM, suggesting an important role of this conserved aromatic residue in ssDNA/RNA interactions. Our study provides the structural basis for further mechanistic studies on this important protein–DNA interaction.

## Graphical abstract

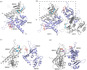

## 1. Introduction

FUBP-interacting repressor (FIR) is a key component of the MYC far upstream element (FUSE) mediated regulatory circuit that tightly regulates MYC transcription. Recruited by FUSE-binding protein 1 (FUBP1), FIR interacts with FUSE [1]. During transcriptional activation, the FUSE sequence unwinds into single-stranded DNA, and the noncoding strand recruits the activator FUBP1. A previous model suggested that the two FUSE strands adopt different conformations upon dsDNA segregation, creating a high-affinity binding site for FUBP1 and two weaker binding sites for the repressor FIR, resulting in a quaternary FIR (2)-FUBP1-FUSE inhibitory complex [2]. The formation of this complex enables the N-terminal repression domain of FIR to target the transcription factor TFIIH helicase, blocking activator-dependent, but not basal, transcription [1,3].

FIR interacts with FUSE through its RNA recognition motifs (RRMs). RRM domains are abundant single-stranded DNA (ssDNA)/RNA binding motifs with a conserved double *β*/*α*/*β* topology. The four anti-parallel β-strands form the central nucleic acid binding surface, which contains two ribonucleoprotein (RNP) consensus sequences. RNP1 is located at the *β*3-strand harbouring eight conserved residues, R/K-G-F/Y-G/A-F/Y-I/L/V-X-F/Y (X is any amino acid), whereas the RNP2 motif is located at the *β*1-strand and is defined by the consensus sequences I/V/L-F/Y-I/V/L-X-Q-L [4]. The aromatic residues within the two RNP motifs are essential for nucleic acid recognition by mediating key stacking interactions with the nucleobases. The importance of the aromatic residues (phenylalanine or tyrosine) in these consensus sequences has been highlighted by single point mutation to alanine, which either greatly reduced binding affinity [5] or in some cases completely abolished nucleic acid binding [6,7].

RRM domains are versatile nucleic acid interaction motifs with varying sequence specificity and a wide range of binding affinities. For instance, hnRNP A1 RRM1 has a strong preference for AGG-containing RNA sequences, which are bound with nanomolar affinity [8]. Sequence specificity of this RRM domain has been demonstrated by a single base substitution of its consensus sequence that reduced the affinity 10-fold [8]. By contrast, the RRM domains of the splicing factor U2AF65 have been shown to generally bind with moderate micromolar affinity to most polypyrimidine-containing sequences, with no preference for particular pyrimidine nucleobases [9].

FIR contains three RRM domains: RRM1 and RRM2 at the N-terminus and the C-terminal RRM3, also known as U2AF homology motif (UHM) [10]. The tandem RRM1 and RRM2 (FIR RRM1-2) are responsible for DNA binding, whereas the function of RRM3, which lacks the usually conserved aromatic residues in its RNP1 and RNP2 motifs, remains unclear [2,11]. Interestingly, RRM3 has been reported to mediate homodimerization or protein-protein interactions instead of binding to ssDNA/RNA [10,12].

FIR RRM1-2 recognizes the FUSE sequence located upstream of the MYC promoter (5′-CCTCGGGATTTTTTATTTTGTGTTATTCC-3′) with low micromolar binding affinity [2,11]. Structural studies on the interaction between FIR RRM1-2 and a FUSE 25-mer nucleotide (nt) revealed that ssDNA bound to the RRM1 domain, however only one nucleobase or a dinucleotide were observed in the binding pocket [11,21]. In addition, it has been proposed that RRM1-2 functions as a dimer, which is formed upon FUSE binding [11]. However, it remains unclear from these earlier observations how binding of a single nucleobase could be sufficient for FUSE interaction. In addition, the predicted functional dimer contradicts an observation from a previous NMR study, which did not detect oligomerization in solution [13].

In this study, we report the high-resolution crystal structure of FIR RRM1-2 in complex with a FUSE 15 nt fragment, demonstrating the ability of RRM1 to recognize multiple regions of FUSE by forming contacts with 2 nt DNA stretches. We also present a comprehensive comparative structural analysis of RRM-ssDNA/RNA interactions, suggesting that two types of RRMs exist that can be classified by the presence of a conserved aromatic amino acid tyrosine (Y-type) or phenylalanine (F-type) in the RNP2 motif. Structural comparison suggests that the differences in this residue mediate distinct intermolecular contacts for nucleic acid recognition.

## 2. Material and methods

### 2.1. Protein expression and purification

The truncated expression construct of FIR RRM1-2 (residues 101–293) was sub-cloned into pNIC28-Bsa4. The recombinant protein with an N-terminal His_6_ tag was overexpressed in phage-resistant *E. coli* BL21 (DE3)-R3-pRARE2 strain. Cells cultured in TB media were initially grown at 37°C, and they were induced with 0.5 mM IPTG at 18°C overnight. Harvested cells were lysed by sonication in a buffer containing 50 mM HEPES, pH 7.5, 500 mM NaCl, 20 mM imidazole, 5% glycerol and 1 mM tris (2-carboxyethyl) phosphine (TCEP). The recombinant protein was first purified by Ni^2+^-affinity chromatography. The His_6_ tag was then removed by TEV protease treatment, and the cleaved protein was passed through Ni^2+^ affinity beads and further purified by size exclusion chromatography using a Superdex S75 column in a buffer containing 25 mM Tris-HCl pH 7.5, 100 mM NaCl. The Y115F mutant was generated by PCR-based site-directed mutagenesis using the forward primer 5′-ATGTGCCGCGTCTTCGTGGGCTCTATCTACTAT’ and the reverse primer 5′-ATAGTAGATAGAGCCCACGAAGACGCGGCACATGAT’. The mutant protein was expressed and purified using the same procedure as for the wild type.

### 2.2. Crystallization, data collection and structure determination

The recombinant protein FIR RRM1-2 and its Y115F mutant were concentrated to approximately 12 mg ml^−1^ and mixed with ssDNA at a 1:2 molar ratio (protein: ssDNA). The mixtures were incubated at 4°C for 3 h prior to size exclusion chromatography to purify the complex. Crystallization was performed using the sitting-drop vapour diffusion method at 20°C. WT-ssDNA complex crystals were obtained in the condition containing 25% w/v PEG 3350, 0.2 M sodium acetate trihydrate and 0.1 M Bis-Tris, pH 5.5. Crystals of the Y115F mutant in complex with ssDNA were obtained in the condition containing 25% w/v PEG 3350 and 0.1 M Bis-Tris, pH 5.5. Crystals were cryoprotected in mother liquor supplemented with 20% ethylene glycol prior to flash cooling in liquid nitrogen.

Diffraction data were collected at beamline X06SA of the Swiss Light Source, Villigen, Switzerland. Data were processed with XDS [14] and scaled with Aimless [15]. All structures were initially solved by molecular replacement using PHASER [16] and the structure of FIR RRM1-2 (PDB ID: 2QFJ). Manual model rebuilding was performed using COOT [17], and the structures were refined using REFMAC5 [18] and PHENIX [19]. The final models were validated with MOLPROBITY [20]. Data collection and refinement statistics are summarized in electronic supplementary material, table S2.

### 2.3. Isothermal titration calorimetry

Isothermal titration calorimetry (ITC) experiments were performed at 20°C in a buffer containing 25 mM HEPES, pH 7.5, 150 mM NaCl and 0.5 mM TCEP using the Affinity ITC instrument (TA Instruments, New Castle, DE). The proteins at 160–240 µM were titrated into the reaction cell containing ssDNA at 40–50 µM using the parameters of 3 µl injection with a total of 30 serial injections. The integrated heat of binding after correction was fitted using an independent single binding site model, based on the manufacturer's protocol, from which thermodynamic binding parameters (ΔH and TΔS), equilibrium association and dissociation constants (*K*_A_ and *K*_D_) and stoichiometry (*n*) were calculated.

## 3. Results

### 3.1. Structural insights into the FIR RRM1-2 interaction with FUSE

**Figure 1.**
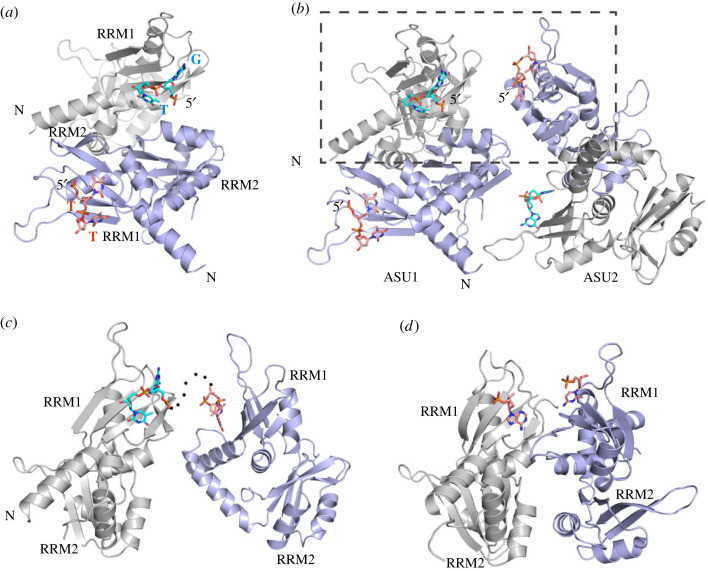
Crystal structure of FIR RRM1-2-FUSE complex. (*a*) Two protein molecules in the asymmetric unit (coloured in grey and light blue, respectively). The bound nucleotide within RRM1 is shown in salmon pink and cyan in stick representations. (*b*) Two neighbouring asymmetric units (ASU1 and ASU2) in the crystal. The dashed box indicates the two FIR RRM1-2 molecules from two neighbouring ASUs that presumably share the same ssDNA: one with 5′-TT (salmon pink) and the other with 5′-GT (cyan). (*c*) The two protein molecules that may interact with the same ssDNA fragment between asymmetric units form no protein-protein contacts, and the ssDNA which contained a central disordered region mediated contacts between these molecules in this crystal form. (*d*) The previously proposed dimeric model of FIR RRM1-2 upon FUSE binding (PDB ID: 2QFJ).

To provide an improved structural model for FUSE recognition, we attempted to crystallize FIR RRM1-2 with ssDNA of various lengths that had their core sequence derived from the FUSE sequence [2,11]. Gratifyingly, we obtained suitable crystals of RRM1-2 in complex with a 15-nt 5′-TTTTTATTTTGTGTT-3′, allowing us to refine the structure at 2.05 Å resolution. The crystals of the FIR-ssDNA complex belonged to the space group *P2_1_2_1_2_1_*, with two protein molecules in the asymmetric unit. In both molecules, only the RRM1 domain bound to the oligonucleotide, while the nucleotide binding surface of RRM2 was occluded by the RRM1 domain, which was consistent with previous structural studies [11,21]. Nonetheless, the two RRM1s in the asymmetric unit were observed to bind to a different sequence: one interacting with a TT and the other with a GT motif (figure 1*a*).

Further structural analysis, which also took the length of the ssDNA and solvent space of crystal packing into consideration, suggested that the bound TT and GT in each protein molecule present in the asymmetric unit did not originate from the same ssDNA (electronic supplementary material, figure S3). However, when applying the crystallographic symmetry, we observed an adjacent neighbouring asymmetric unit in which the RRM1 bound to the corresponding second binding site of ssDNA (figure 1*b*). Thus, in the arrangement in the crystal, it is likely that one oligonucleotide bridged two RRM1-2 domains from two neighbouring asymmetric units, which however did not form any protein-protein contacts (figure 1*c*). This binding mode was in a stark contrast to the models of the RRM-ssDNA dimer postulated previously [11,21] in which two FIR molecules that bound different parts of the FUSE ssDNA formed an intermolecular protein domain interface (figure 1*d*). At least in the observed crystal structure, our structural model suggested that each FIR RRM domain bound ssDNA independently, yet multiple recruitments of the protein may occur on a single FUSE sequence. This observation is in agreement with a previous NMR study reporting a functioning monomer in solution [13].

### 3.2. Molecular basis of FIR RRM1-2–FUSE recognition

**Figure 2.**
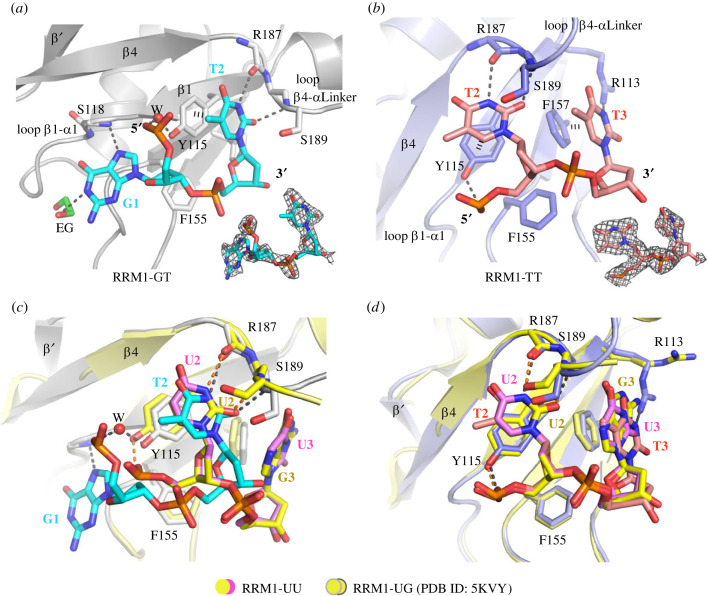
Intermolecular interactions between FIR RRM1 and ssDNA. Interactions of RRM1 with ‘GT’ (*a*) and ‘TT’ motifs (*b*). Guanine was observed to form a hydrogen bond with the solvent ethylene glycol (EG, coloured in green). Hydrogen bonds are shown as dashed lines. *F*_o_–*F*_c_ omit electron density maps contoured at 3.0 *σ* for the bound ssDNA are displayed at the bottom. (*c*) Structural comparison of the interaction of the RRM1-GT complex and the previously published FIR RRM1-UU and RRM1-UG complexes (PDB ID: 5KVY). (*d*) Structural comparison of the RRM1-TT complex and the previously published RRM1-UU and RRM1-UG complexes (PDB ID: 5KVY).

The structure of the RRM1-2 interaction with FUSE revealed that RRM1 engaged its central β-sheet surface, the conserved aromatic residues in RNP motifs, and residues located in the flexible loop region between the *β*1-*α*1 and *β*4-linker helix for nucleotide binding (figure 2*a,b*). However, the two regions of FUSE ssDNA that complexed with the RRM domains were accommodated in different binding sites and exerted distinct protein-ssDNA interactions. In the GT ssDNA sequence, the 5′-end guanine in position 1 was adjacent to the *β*1-α1 loop, forming a direct interaction between the nucleobase and the backbone amide of Ser118. In addition, the 5′-end phosphate group formed a water-mediated hydrogen bond with Tyr115 in strand *β*1 (figure 2*a*). The thymine located at nucleotide binding position 2 had its nucleobase sandwiched between the side chains of Tyr115 and Ser189, resulting in the formation of two hydrogen bonds: one between the base nitrogen atom N3 and the carbonyl backbone of Arg187, and the other between the 2-carbonyl group and the backbone amide nitrogen atom of Ser189. The scenario was different for the TT stretch. Although residual electron density was observed at position 1, this density was too poor to confidently model the nucleotide. The binding mode of the thymine at position 2 resembled that of the GT thymine, including the stacking of the nucleobase with Tyr115 and Ser189 as well as the two hydrogen bonds with the backbones of Arg187 and Ser189 (figure 2*b*). However, the phosphate backbone formed a hydrogen bond with the hydroxyl group of Tyr115 instead of facing to the solvent region as observed in the RRM1-GT complex. The thymine at position 3 formed a π-stacking interaction with Phe157 on the *β*3-strand and engaged in a hydrogen bond with Arg113 (figure 2*b*).

We next compared our complex structures with available complexes of FIR RRM1-UU and RRM1-UG (PDB ID: 5KVY) [21]. Interestingly, while the configuration of TT observed in our structure aligned well with the dinucleotides UU and UG (figure 2*d*), distinct phosphate backbone configurations were evident for GT, resulting in a different orientation of the DNA and, therefore, contacts with the protein (figure 2*c*). Apart from differences for the nucleotides, structural comparison also highlighted slight differences in the conformation of the protein, for example affecting a number of residues located in the binding pocket, such as Ser189 and Arg113, as well as an extended *β*4-strand that potentially induced an extra β-strand, *β*’, after helix *α*2 (figure 2*c,d*), highlighting the versatility of FIR RRM1-2 in nucleic acid recognition.

### 3.3. Diverse nucleic acid binding configurations in different RRM-ssDNA/RNA interactions

**Figure 3.**
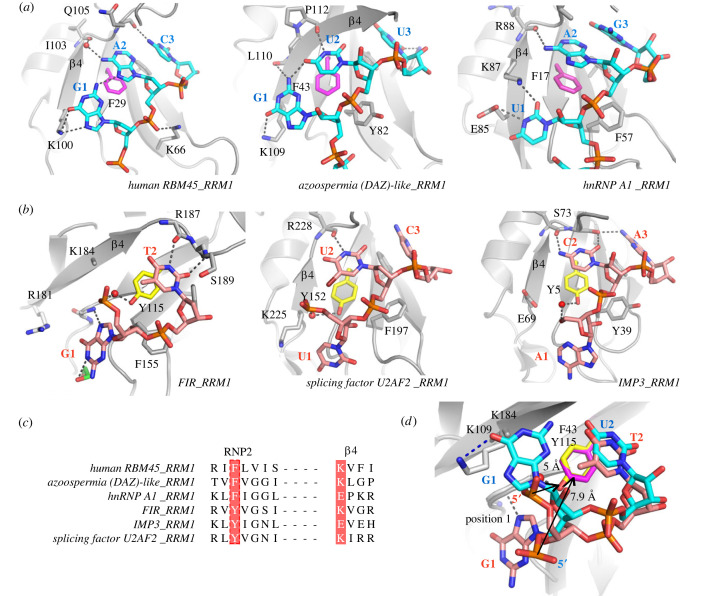
Comparative structural analyses of ssDNA/RNA binding modes in diverse RRMs. (*a*) The ssDNA/RNA recognition in human RBM45 (PDB ID: 7CSZ), DAZL (PDB ID: 2XS5), and hnRNP A1 (PDB ID: 5MPG), all of which share a phenylalanine residue in their RNP2 motifs. Hydrogen bonds are shown as dashed lines. (*b*) The ssDNA/RNA recognition of FIR, splicing factor U2AF2 (PDB ID: 6XLW), and IMP3 (PDB ID: 6GX6), all of which share a tyrosine residue in their RNP2 motifs. (*c*) Sequence alignment of the RNP2 motif and *β*4 of these selected RRMs. The conserved aromatic residues in RNP2 and the positively/negatively charged residue in the *β*4 are highlighted. (*d*) Superimposition of the bound nucleotide in position 1 of ‘Y-type’ (FIR; salmon pink) and ‘F-type’ (DAZL; cyan) RRMs. The distance between the tyrosine/phenylalanine residue and the phosphorus atom of the bound ssDNA in position 1 is labelled. Hydrogen bonds are shown as dashed lines.

The observation of different binding configurations of nucleotide in FIR RRM1 prompted us to investigate whether the observed 5′-end nucleotide binding configuration resembled interactions observed in other RRM-ssDNA/RNA complexes. We randomly chose five structures of other nucleic acid-bound RRM1 motifs, including human RBM45 [22] (PDB ID: 7CSZ), DAZL (azoospermia (DAZ)-like) [23] (PDB ID: 2XS5), hnRNP A1 [8] (PDB ID: 5MPG), U2AF2 [24] (PDB ID: 6XLW), and IMP3 [25] (PDB ID: 6GX6), for comparison (figure 3*a*,*b*). Structural superimposition revealed that while the configurations of the nucleotide in position 2 and position 3 are generally conserved, including the orientations of the nucleobase and the backbone phosphate group, significantly different orientations of the bound nucleobase in position 1 between RRMs complexes were observed. In RBM45, DAZL and hnRNP A1, the bound nucleobases were oriented towards to the core β-sheet and formed direct hydrogen bonds with residues on *β*4 (figure 3*a*). By contrast, these nucleobases were shifted away from the β-sheet surface in U2AF2 and IMP3 (figure 3*b*), leading to a lack of interaction of the 5′-end nucleobase with the conserved charged amino acid in *β*4, typically either a lysine or a glutamate (figure 3*c*) that normally engaged in a direct contact with the nucleobases in the counterpart RRMs. Notably, in our FIR RRM1-GT structure, we observed that despite the 5′-end guanine forming a direct hydrogen bond with the protein, it was positioned far away from *β*4 and mainly interacted with the surface formed by loops *β*1-α1 and *β*2-β3. Thus, distinct interactions of the 5′-end nucleotide and therefore unique nucleic acid binding configurations were evident in different RRM-ssDNA/RNA complexes.

Detailed structural comparison suggested that the different conserved aromatic residue present in the RNP2 motif might contribute to the distinct orientations of the bound oligonucleotides. The presence of the hydroxyl group in the tyrosine side chain enabled a water-mediated hydrogen bond with the nucleotide backbone, leading to stabilization of the nucleotide backbone and positioning of the interacting nucleotide close to the β-sheet surface. This binding mode is exemplified by FIR RRM1 in which the phosphorus atom of the 5′-end nucleotide was located at a distance of approximately 5 Å from Tyr115. This resulted in an outward trajectory of the nucleobase in position 1, away from the protein (figure 3*d*). By contrast, the phosphate backbones of the nucleotides were typically projected further away from the core β-sheet when binding to the RRMs with a phenylalanine at the RNP2 motif, shown for example by an approximately 8 Å distance between the 5′-end phosphorus atom and Phe29 in the DAZL RRM1-GUU complex (figure 3*d*). This configuration enabled most likely the placement of the nucleobase in position 1 at the β-sheet surface, enabling intermolecular hydrogen bonds with the *β*4 residues.

**Figure 4.**
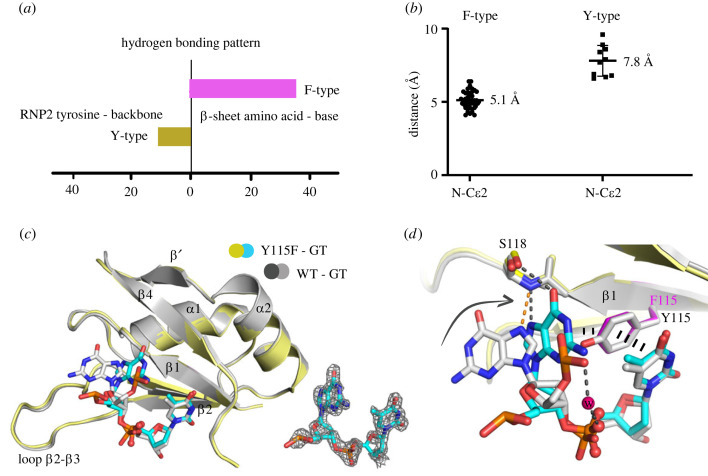
The aromatic residue in RNP2 is associated with two distinct nucleic acid binding modes. (*a*) The number of the Y- and F-type RRM–nucleic acid structures in the PDB that share the type-associated binding modes for the interaction of the nucleobase at the 5′-end. (*b*) Distances between the C*ε*2 atom of the aromatic ring in *β*1 (i.e. the C*ε* atom facing *β*4) and the sugar-linked nitrogen of the base at position 1 in F-type and Y-type RRMs in the PDB (see also electronic supplementary material, table S1). This distance was much shorter in the F-type (average distance of 5.1 Å) than in the Y-type (average distance of 7.8 Å), highlighting a different orientation of the nucleotide at position 1 in the two subgroups. (*c*) Structural superimposition of Y115F mutant (coloured in yellow and cyan) and wild-type (WT, coloured in grey) FIR RRM1 binding to ‘GT’ ssDNA. F_o_-F_c_ omit electron density maps contoured at 3.0 *σ* for the nucleotides in the Y115F mutant complex are shown at bottom. (*d*) The 5′-end guanine is flipped towards the β-sheet surface when replacing the tyrosine in *β*1 by phenylalanine, leading to the formation of an additional side chain-nucleobase hydrogen bond.

We next investigated whether the correlation between the specific aromatic residue in the RNP2 motif and the distinct hydrogen bonding patterns observed among the selected RRMs could be generalized for other RRMs. We therefore analysed the structures of nucleotide-bound RRMs in the PDB database that satisfied the conditions of (i) containing at least 2 nucleotides occupying the position 1 and 2 in the complex, and (ii) a resolution of at least 2.8 Å. A total of 56 structures were inspected: 42 contained a phenylalanine residue in the RNP2 motif, and we referred to them as F-type RRMs; 11 contained a tyrosine, which in analogy were termed Y-type RRMs (figure 4*a*; electronic supplementary material, table S1); and the other 3 harboured instead a histidine residue at this position (electronic supplementary material, table S1 and figure S1) [26,27]. Comparative structural analyses revealed that 35 of 42 F-type RRMs shared the binding mode as depicted in figure 3*a* in which the nucleobase in position 1 was oriented towards the β-strands and formed direct or water-mediated hydrogen bonds with *β*4 residues. 4 of 42 F-type RRMs were observed to use the residues in loop *β*1-α1 instead of *β*4 for the 5′-end nucleobase interaction (electronic supplementary material, figure S2*a*,*b*). By contrast, the equivalent nucleobases were consistently positioned further away from the β-strands in the Y-type RRMs. In addition, 10 of 11 Y-type RRMs formed a hydrogen bond between the tyrosine and the nucleotide backbone, either in position 1 or in position 2. The distinct binding modes were also reflected in the distance between the nucleobase in position 1 and the conserved aromatic residue. The average distance between the C*ε*2 atom of the aromatic ring (i.e. the C*ε* atom facing *β*4) and the sugar-linked nitrogen of the base at position 1 was 7.8 Å for the Y-type RRMs compared with an average distance of only 5.1 Å in the F-type RRMs (figure 4*b*; electronic supplementary material, table S1). This structural analysis therefore suggested a potential role of the RNP2 aromatic residue in influencing nucleic acid binding configurations.

### 3.4. The aromatic residue in the RNP2 motif regulates nucleic acid binding modes

To further investigate whether the aromatic residue within RNP2 might be responsible for the orientation of the nucleobase at the 5′-end that led to distinct binding modes, we generated a FIR RRM1-2 Y115F mutant and cocrystallized this mutant with the same 15-nt ssDNA that was used for determining the structure of the wild-type (WT) complex. Crystals of the Y115F mutant were isomorphous to those of the WT, and the structure of the mutant was determined at 1.65 Å resolution. As in the WT complex, the two protein molecules in the asymmetric unit were observed to bind one ssDNA each, with one protein molecule binding to GT and the other one interacting with TT at the same binding sites.

Because we were interested in the intermolecular interactions between protein and the bound 5′-end nucleotide, we superimposed WT and mutated RRM1-GT complex structures for comparison. Structural superimposition revealed that while the protein conformation was highly conserved, with an RMSD (C_α_) of 0.25 Å, the bound ssDNA had undergone significant conformational changes, specifically the orientation of the 5′-end nucleobase, leading to distinct intermolecular contacts (figure 4*c*,*d*). The replacement of the tyrosine by a phenylalanine in RNP2 abolished the hydrogen bond formation between the tyrosine and ssDNA backbone, which enabled the guanine base to flip towards the β-strands. This orientation facilitated the formation of hydrogen bonds between the guanine carbonyl group and N7 atom with the side chain and backbone of Ser118, respectively. In addition, the amino group of the base formed a water-mediated intramolecular hydrogen bond with the backbone phosphate group (figure 4*d*). The flipped guanine formed a π-stacking interaction with Phe115, further stabilizing the observed binding mode. Such a configuration and its associated intermolecular interactions, including the hydrogen-bond network as well as the π-stacking with the RNP2 aromatic residue, were observed in several F-type RRMs that specifically recognize guanosine in position 1, which was distinct from the binding mode of Y-type RRMs (electronic supplementary material, figure S2*c*). By contrast, the recognition of the thymine in position 2 was well conserved (figure 4*d*). Taken together, our structural study of the Y115F mutant suggested therefore an influence of the aromatic residue in RNP2 on the orientation of the nucleobase in position 1.

### 3.5. The role of the RNP2 aromatic residue in determining sequence preference and binding affinity

**Figure 5.**
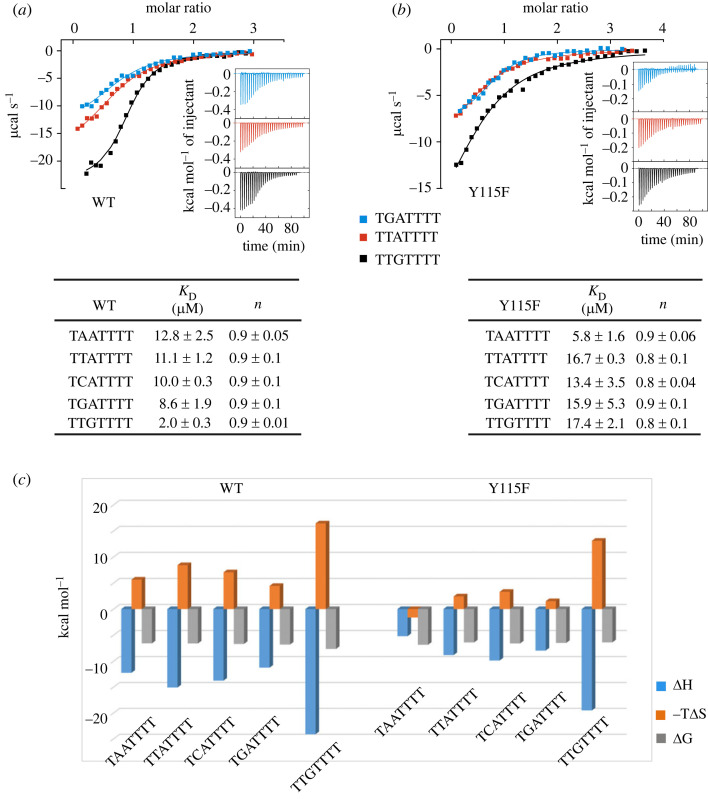
ITC measurement comparing wild type and Y115F mutant FIR RRM1 binding to diverse ssDNAs. Shown are representative ITC data for the interaction of wild type (WT; *a*) and Y115F mutant (*b*) RRM1-2 with ssDNA fragments TTATTTT, TGATTTT and TTGTTTT. The integrated heat of binding with independent binding fitting are depicted in the main graph, with the insets showing the binding isotherms of the raw titration heat. ITC binding constants (*K*_D_) are shown in the tables below. The ITC errors were calculated from averaging results for ITC duplicates (*c*) Calculated thermodynamic parameters for the binding of WT or Y115F mutant to the ssDNA fragments.

Next, we were interested in the influence of the RNP2 aromatic residue on the affinity and specificity of ssDNA binding. We used isothermal titration calorimetry (ITC) to compare the interactions of the FIR Y115F mutant and WT with five random thymine-rich ssDNA sequences with sequence variations in the 5′-end half (TAATTTT, TTATTTT, TCATTTT, TGATTTT and TTGTTTT). We observed that the mutation indeed affected the affinities of the tested ssDNA sequences (figure 5*a,b*). WT showed a 4-6-fold binding preference for the ssDNA TTGTTTT sequence among the test set fragments, with a measured dissociation constant, *K*_D_, of 2 µM. By contrast, the Y115F mutant showed only a slight binding preference for ssDNA TAATTTT, exhibiting a 2-fold higher affinity for this sequence than for the other four ssDNA fragments tested. Notably, both WT and mutant showed distinct thermodynamic signatures upon binding ssDNA TTGTTTT, which was characterized by an increased favourable enthalpy that was opposed by a large entropic penalty (figure 5*c*). This may be due to an intrinsic property of this particular ssDNA as well as additional inter/intramolecular contacts leading to lower flexibility of both protein and ssDNA in the bound state.

Overall, the Y115F mutant and the WT protein showed comparable ssDNA binding affinities, with a *K*_D_ around 6 and 2 µM for their preferred sequences, respectively, while the other four ssDNA fragments bound with a *K*_D_ ranging between 10 and 20 µM (figure 5*a,b*). However, it is interesting to note that the TTGTTTT fragment had only one base difference compared with the TTATTTT FUSE sequence. In this case, a single purine base substitution from A to G led to a remarkably improved binding affinity in FIR RRM1 (*K_D_* of 11 versus 2 µM, respectively), whereas the Y115F mutant bound both sequences with virtually the same affinity, indicating that the tyrosine in RNP2 of FIR RRM1 might play a crucial role in selective FUSE recognition. Since the Y115F mutation in FIR RRM1 did not increase the sequence specificity nor binding affinity, a more complex mechanism might be responsible for determining the two distinct binding modes. The different binding preference as well as the diverse thermodynamic signatures of FIR Y115F and WT (figure 5*c*) are in agreement with the postulated diverse interaction mechanisms when comparing the binding of WT (tyrosine-containing) and mutant (phenylalanine-containing) RRM domains to the selected ssDNA sequences.

## 4. Discussion

We have provided a high-resolution model of the FIR RRM1-2-FUSE interaction and presented a comprehensive structural analysis of RRM-ssDNA/RNA interactions. We observed distinct binding modes of different RRM-ssDNA interactions and no binding-induced RRM1-2 protein-protein interaction as postulated previously [11,21]. Together with ITC binding data, our structural data demonstrated that FIR RRM1 has the potential to interact with diverse ssDNA sequences, consistent with previous data that reported on the low specificity of this protein [11]. Along with the previous structural studies [11,21], our crystal structures, which present different binding modes of the two bound dinucleotides, extend the repertoire of structural models for nucleotide interactions in FIR. The structural plasticity observed highlights the versatility of FIR RRMs in accommodating diverse oligonucleotide sequences, in agreement with published binding studies [11,21]. Overall, our observations demonstrate the dynamic nature of the RRM1–FUSE interface, which is consistent with an earlier study that suggested RRM as a versatile ssDNA/RNA-binding protein module contributing to a wide spectrum of nucleic acid sequence recognition [4].

Intriguingly, our comparative structural analysis defined two major groups of RRMs based on different types of the conserved aromatic residue in the RNP2 motif, which potentially play a role in regulating nucleotide binding modes: the tyrosine- (Y-type) and phenylalanine-containing (F-type) RRMs. Distinct nucleic acid binding modes, especially at the 5′-end position, were observed among these two types of RRMs. We hypothesized that the tyrosine side chain in the Y-type can engage in additional contacts with the nucleic acid backbone that is generally bound in closer proximity to the β-sheet core when compared with F-type structural models. Using FIR as a model, we observed indeed that substitution of the RNP2 tyrosine to phenylalanine significantly altered the orientation of the 5′-end nucleobase, facilitating distinct intermolecular contacts, while the overall structure of the protein was well conserved. Our ITC binding data also suggested that Y115F mutation led to a change in binding behaviours of the tested ssDNA fragments, further supporting the role of this aromatic residue in regulating nucleic acid recognition.

Our comparative structural analyses suggest that nucleotide recognition in RRMs is a complex mechanism and involves many residues at the binding interface. This is highlighted also by a recent study showing that the nature of the residues at the N terminus of *β*4 is crucial for nucleotide preference through the formation of direct contacts with different bases [28]. In addition, dynamics of the conserved aromatic residue in the RNP2 motif involving its side-chain conformational changes could modulate the binding affinities of nucleotides [29,30]. The latter is consistent with our observation of two different binding nucleotide configurations in Y- or F-type RRMs and the altered nucleic acid recognition in the FIR Y115F mutant. Taken together, our comprehensive structural and biochemical data offer not only detailed insights into nucleic acid binding in FIR but also revealed an important role of the conserved aromatic residue in the RNP2 motif in modulating the orientation of bound oligonucleotides, thus providing a structural basis for future mechanistic studies on nucleotide recognition in RRMs.

## Data availability

Atomic coordinates and structure factors for the reported crystal structures have been deposited in the Protein Data Bank (PDB) under accession codes 7Q8A and 7Z3X.

Additional structural data and analyses are provided in the electronic supplementary material [31].

## References

[1] Liu J, He L, Collins I, Ge H, Libutti D, Li J, Egly JM, Levens D. 2000 The FBP interacting repressor targets TFIIH to inhibit activated transcription. Mol. Cell **5**, 331-341. (10.1016/S1097-2765(00)80428-1) 10882074

[2] Hsiao HH, Nath A, Lin CY, Folta-Stogniew EJ, Rhoades E, Braddock DT. 2010 Quantitative characterization of the interactions among c-myc transcriptional regulators FUSE, FBP, and FIR. Biochemistry **49**, 4620-4634. (10.1021/bi9021445) 20420426

[3] Liu J, Kouzine F, Nie Z, Chung HJ, Elisha-Feil Z, Weber A, Zhao K, Levens D. 2006 The FUSE/FBP/FIR/TFIIH system is a molecular machine programming a pulse of c-myc expression. EMBO J. **25**, 2119-2130. (10.1038/sj.emboj.7601101) 16628215 PMC1462968

[4] Maris C, Dominguez C, Allain FH. 2005 The RNA recognition motif, a plastic RNA-binding platform to regulate post-transcriptional gene expression. FEBS J. **272**, 2118-2131. (10.1111/j.1742-4658.2005.04653.x) 15853797

[5] Sickmier EA, Frato KE, Shen H, Paranawithana SR, Green MR, Kielkopf CL. 2006 Structural basis for polypyrimidine tract recognition by the essential pre-mRNA splicing factor U2AF65. Mol. Cell **23**, 49-59. (10.1016/j.molcel.2006.05.025) 16818232 PMC2043114

[6] Maegawa S, Yamashita M, Yasuda K, Inoue K. 2002 Zebrafish DAZ-like protein controls translation via the sequence ‘GUUC’. Genes Cells **7**, 971-984. (10.1046/j.1365-2443.2002.00576.x) 12296827

[7] Teplova M, Farazi TA, Tuschl T, Patel DJ. 2016 Structural basis underlying CAC RNA recognition by the RRM domain of dimeric RNA-binding protein RBPMS. Q. Rev. Biophys. **49**, e1. (10.1017/S0033583515000207) 26347403 PMC4783296

[8] Beusch I, Barraud P, Moursy A, Clery A, Allain FH. 2017 Tandem hnRNP A1 RNA recognition motifs act in concert to repress the splicing of survival motor neuron exon 7. Elife **6**, e25736. (10.7554/eLife.25736) 28650318 PMC5503513

[9] Jenkins JL, Agrawal AA, Gupta A, Green MR, Kielkopf CL. 2013 U2AF65 adapts to diverse pre-mRNA splice sites through conformational selection of specific and promiscuous RNA recognition motifs. Nucleic Acids Res. **41**, 3859-3873. (10.1093/nar/gkt046) 23376934 PMC3616741

[10] Page-McCaw PS, Amonlirdviman K, Sharp PA. 1999 PUF60: a novel U2AF65-related splicing activity. RNA **5**, 1548-1560. (10.1017/S1355838299991938) 10606266 PMC1369877

[11] Crichlow GV et al. 2008 Dimerization of FIR upon FUSE DNA binding suggests a mechanism of c-myc inhibition. EMBO J. **27**, 277-289. (10.1038/sj.emboj.7601936) 18059478 PMC2206118

[12] Kielkopf CL, Lucke S, Green MR. 2004 U2AF homology motifs: protein recognition in the RRM world. Genes Dev. **18**, 1513-1526. (10.1101/gad.1206204) 15231733 PMC2043112

[13] Cukier CD, Hollingworth D, Martin SR, Kelly G, Diaz-Moreno I, Ramos A. 2010 Molecular basis of FIR-mediated c-myc transcriptional control. Nat. Struct. Mol. Biol. **17**, 1058-1064. (10.1038/nsmb.1883) 20711187 PMC2964917

[14] Kabsch W. 2010 Xds. Acta Crystallogr. D Biol. Crystallogr. **66**(Pt 2), 125-132. (10.1107/S0907444909047337) 20124692 PMC2815665

[15] Evans PR, Murshudov GN. 2013 How good are my data and what is the resolution? Acta Crystallogr. D Biol. Crystallogr. **69**(Pt 7), 1204-1214. (10.1107/S0907444913000061) 23793146 PMC3689523

[16] McCoy AJ. 2017 Acknowledging errors: advanced molecular replacement with phaser. Methods Mol. Biol. **1607**, 421-453. (10.1007/978-1-4939-7000-1_18) 28573584

[17] Emsley P. 2017 Tools for ligand validation in Coot. Acta Crystallogr. D Struct. Biol. **73**(Pt 3), 203-210. (10.1107/S2059798317003382) 28291755 PMC5349432

[18] Murshudov GN, Skubak P, Lebedev AA, Pannu NS, Steiner RA, Nicholls RA, Winn MD, Long F, Vagin AA. 2011 REFMAC5 for the refinement of macromolecular crystal structures. Acta Crystallogr. D Biol. Crystallogr. **67**(Pt 4), 355-367. (10.1107/S0907444911001314) 21460454 PMC3069751

[19] Liebschner D et al. 2019 Macromolecular structure determination using X-rays, neutrons and electrons: recent developments in Phenix. Acta Crystallogr. D Struct. Biol. **75**(Pt 10), 861-877. (10.1107/S2059798319011471) 31588918 PMC6778852

[20] Williams CJ et al. 2018 MolProbity: More and better reference data for improved all-atom structure validation. Protein Sci. **27**, 293-315. (10.1002/pro.3330) 29067766 PMC5734394

[21] Hsiao HT, Crichlow GV, Murphy JW, Folta-Stogniew EJ, Lolis EJ, Braddock DT. 2020 Unraveling the mechanism of recognition of the 3’ splice site of the adenovirus major late promoter intron by the alternative splicing factor PUF60. PLoS ONE **15**, e0242725. (10.1371/journal.pone.0242725) 33253191 PMC7703929

[22] Chen X, Yang Z, Wang W, Qian K, Liu M, Wang J, Wang M. 2021 Structural basis for RNA recognition by the N-terminal tandem RRM domains of human RBM45. Nucleic Acids Res. **49**, 2946-2958. (10.1093/nar/gkab075) 33577684 PMC7968997

[23] Jenkins HT, Malkova B, Edwards TA. 2011 Kinked beta-strands mediate high-affinity recognition of mRNA targets by the germ-cell regulator DAZL. Proc. Natl Acad. Sci. USA **108**, 18 266-18 271. (10.1073/pnas.1105211108) 22021443 PMC3215079

[24] Maji D, Glasser E, Henderson S, Galardi J, Pulvino MJ, Jenkins JL, Kielkopf CL. 2020 Representative cancer-associated U2AF2 mutations alter RNA interactions and splicing. J. Biol. Chem. **295**, 17 148-17 157. (10.1074/jbc.RA120.015339) PMC7863893 33020180

[25] Jia M, Gut H, Chao JA. 2018 Structural basis of IMP3 RRM12 recognition of RNA. RNA **24**, 1659-1666. (10.1261/rna.065649.118) 30135093 PMC6239170

[26] Auweter SD, Fasan R, Reymond L, Underwood JG, Black DL, Pitsch S, Allain FH. 2006 Molecular basis of RNA recognition by the human alternative splicing factor Fox-1. EMBO J. **25**, 163-173. (10.1038/sj.emboj.7600918) 16362037 PMC1356361

[27] Upadhyay SK, Mackereth CD. 2020 Structural basis of UCUU RNA motif recognition by splicing factor RBM20. Nucleic Acids Res. **48**, 4538-4550. (10.1093/nar/gkaa168) 32187365 PMC7192616

[28] Roca-Martinez J, Dhondge H, Sattler M, Vranken WF. 2023 Deciphering the RRM-RNA recognition code: a computational analysis. PLoS Comput. Biol. **19**, e1010859. (10.1371/journal.pcbi.1010859) 36689472 PMC9894542

[29] Vitali J, Ding J, Jiang J, Zhang Y, Krainer AR, Xu RM. 2002 Correlated alternative side chain conformations in the RNA-recognition motif of heterogeneous nuclear ribonucleoprotein A1. Nucleic Acids Res. **30**, 1531-1538. (10.1093/nar/30.7.1531) 11917013 PMC101846

[30] Diarra Dit Konte N, Krepl M, Damberger FF, Ripin N, Duss O, Sponer J, Allain FH. 2017 Aromatic side-chain conformational switch on the surface of the RNA Recognition Motif enables RNA discrimination. Nat. Commun. **8**, 654. (10.1038/s41467-017-00631-3) 28935965 PMC5608764

[31] Ni X, Joerger AC, Chaikuad A, Knapp S. 2023 Structural insights into a regulatory mechanism of FIR RRM1–FUSE interaction. *Figshare*. (10.6084/m9.figshare.c.6662119) PMC10229228 37253421

